# Influence of social distancing during the COVID-19 pandemic on physical activity in children: A scoping review of the literature

**DOI:** 10.1016/j.jesf.2021.04.002

**Published:** 2021-05-11

**Authors:** Kenji Yomoda, Shohei Kurita

**Affiliations:** a Nagoya Gakuin University, Japan; b St. Catherine University, Japan

**Keywords:** Child health, COVID-19, Exercise, Scoping review

## Abstract

**Background/objectives:**

There is an urgent concern about physical inactivity among children caused by recent restrictions for containing the spread of the novel coronavirus disease (COVID-19). This scoping review aims to identify the impact of the pandemic and related factors on children's physical activity (PA).

**Methods:**

Twenty-one studies published in 2020 that evaluated PA among children under the age of 18 were extracted from electronic databases. The survey contexts, samples, PA measurement methods, and main findings of each study were categorized and summarized.

**Results:**

The review yielded the following results: 1) the PA of children and adolescents mostly decreased; 2) the decrease in PA was more prevalent in boys and older children and adolescents; 3) the decrease in PA was less prevalent in children who live in detached houses, houses with more space, rural areas, and with more family members; and 4) parental support and consideration of location and activity types may help children maintain or increase their PA during the pandemic.

**Conclusion:**

This review shows a decline in PA among children and raises concerns about the pandemic's impact on physical and mental health. Declines in PA appear greater among children who participate in organized team sports and those with limited space and opportunity for habitual PA at home or in their neighborhood. Public support for children and families should consider these findings to prevent the negative effects of physical inactivity.

## Background

The novel coronavirus disease (COVID-19) has been spreading worldwide, and social restrictions for containing the spread of the pandemic such as lockdowns or quarantines have had a profound impact on people's lives. By April 2020, schools had been closed in 195 countries, affecting 1.6 billion children (95% of the world's school-aged children), and closures lasted an average of 3.5 months of an academic year.^1^

The social restrictions have raised concerns about potential risks to children's health, including physical inactivity, sedentary behaviors, sleep patterns, screen exposures, and mental health problems. In fact, studies have reported sleep disturbance and increased levels of depression and anxiety among children and adolescents because of the COVID-19 pandemic.^2^, ^3^, ^4^ Additionally, a decline in physical activity (PA) among children may be associated with school closures, cancellation of club practices, and stay-at-home restrictions.^5^, ^6^, ^7^ Childhood regular PA habits are vital for acquiring fundamental movement skills, promoting physical, and psychological development and are also important in that they are associated with health-related lifestyle habits, obesity, and cardiovascular diseases in adulthood.^8^^,^^9^

Caputo and Reichert^10^ reviewed studies examining the impacts on PA during the COVID-19 pandemic and reported a worldwide decrease in PA and associated negative effects on mental health. However, only nine of the 41 studies reviewed focused on children, and the studies' findings related to children were not discussed. López-Valenciano et al.^11^ reviewed studies examining the PA of university students during the pandemic and found that nine out of ten reported a significant decrease in PA. They also noted that students who were sufficiently active before the pandemic remained sufficiently active during the confinements, despite reductions in their overall PA levels. Therefore, although several studies have measured the PA of children during the pandemic, a comprehensive review of these studies has yet to be conducted. Accordingly, this study aims to review the impact of the COVID-19 pandemic and related factors on children's PA. The outcome of the review will help healthcare workers, researchers, and schoolteachers consider how public support for children and families can help prevent the negative effects of inactivity.

## Methods

### Research design

Research articles that measured changes in children's PA during the COVID-19 pandemic were reviewed. As the global pandemic is ongoing, a scoping review method was used to create quickly an overview of existing studies.^12^ The scoping review checklist (PRISMA-ScR)^13^ was used to describe the review procedure.

### Study selection

**Figure 1:**
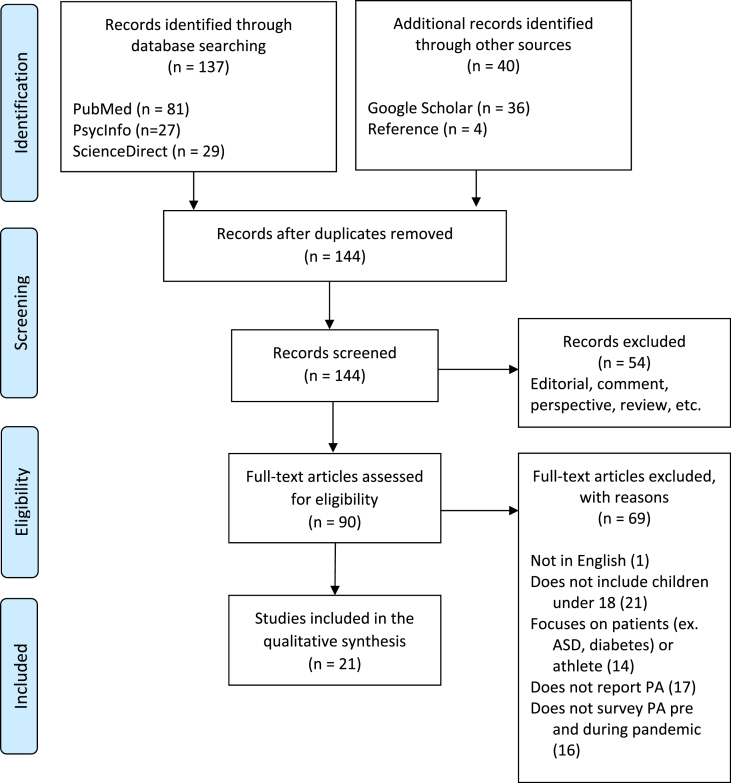
Overview of the study selection process.

On January 6, 2021, database searches were conducted on PubMed, EBSCO (PsycInfo), and ScienceDirect using the criteria outlined in Table 1. Additional articles were acquired using Google Scholar and the reference lists of the extracted articles. After excluding duplicates, the titles and abstracts of 144 studies were screened. In this primary screening, original research articles were included, and other types of articles (e.g., editorials, comments, perspectives, or reviews) were excluded. In the secondary eligibility screening, during which the full study texts were used, articles were selected based on the following criteria: a) written in English, b) includes children under the age of 18 as participants (or they are included as a target group), c) does not target a specific sample (e.g., patients or junior athletes), d) measures PA levels or time, and e) surveys changes in PA before and during the pandemic (those that measured only PA during the pandemic were excluded). The study selection process and the number of excluded papers are shown in Fig. 1. Finally, 21 studies were included in the analysis.

**Table 1:** Database search criteria.

Database	Search criteria
PubMed	COVID [Title/Abstract]) AND (physical activity [Title/Abstract]) AND (child∗[Title/Abstract] OR youth∗[Title/Abstract] OR adolesc∗[Title/Abstract]
EBSCO (PsycInfo)	(COVID or coronavirus or covid-19 or sars-cov-2) AND physical activity AND (children or adolescents or youth or child or teenager)
ScienceDirect	COVID AND physical activity AND (child OR children OR youth OR youths OR adolescent OR adolescents OR adolescence)

### Data analysis and synthesis

The research contexts and main findings of each study were charted according to the following: a) author, year, and country, b) survey period, c) age and number of samples, d) data collection methods, and e) main findings. For data collection methods, PA measurement methods and comparison strategies (baseline and follow-up/recall/perceived changes) between before and during the pandemic were categorized. To identify characteristics and provide an overview of the studies, demographic characteristics of the research contexts and methods were aggregated. Findings about significant changes in PA, screen time, sedentary behaviors, and sleep duration during the pandemic were compiled. The factors each study described to be associated with PA were also categorized: age, gender, living environment, and parental factors. The first and second authors conducted the analyses independently, and any discrepancies were resolved through discussion.

## Results

An overview of the demographic characteristics and the findings of each study are presented in Table 2, Table 3, and Table 4, and a descriptive list of the research context and main findings of each study are charted in Appendix Table A.

**Table 2:** Summary of demographic characteristics.

Region^a^	Number of cases		Articles
Europe	11		^19^,^14^,^15^,^16^,^17^,^18^,^20^,^21^,^22^,^23^,^24^
Americas	7		^25^,^26^,^27^,^28^,^21^,^29^,^30^
South-East Asia	2		^31^,^32^
Eastern Mediterranean	1		^33^
Western Pacific	1		^34^
Africa	0		–
**Age group**			
Preschool	7		^25^,^14^,^16^,^19^,^20^,^29^,^22^
Childhood	11		^26^,^14^,^16^,^27^,^17^,^28^,^30^,^20^,^21^,^29^,^22^
Adolescent	17		^33^,^14^,^15^,^31^,^16^,^27^,^17^,^28^,^30^,^34^,^18^,^20^,^21^,^22^,^23^,^32^,^24^
**Sample size**			
n < 100	2		^33^,^19^
100 ≤ n < 500	4		^26^,^27^,^17^,^23^
500 ≤ n < 1000	6		^15^,^16^,^34^,^21^,^29^,^24^
n ≥ 1000	9		^25^,^14^,^31^,^28^,^30^,^18^,^20^,^22^,^32^
**PA measurement**			
Online questionnaire	21		
PAQ-A		4	^33^,^15^,^23^,^24^
IPAQ		3	^31^,^21^,^32^
PACE+		2	^34^,^18^
Others		12	^25^,^26^,^14^,^16^,^27^,^17^,^28^,^30^,^19^,^20^,^29^,^22^
Measuring devices	2		
Tri-axial accelerometer		1	^19^
Smartphone sensors		1	^34^
**Data comparison**			
Baseline & follow-up	8		^33^,^15^,^17^,^34^,^19^,^22^,^23^,^24^
Recall before pandemic	7		^25^,^14^,^31^,^16^,^20^,^21^,^32^
Perceived changes	6		^26^,^27^,^28^,^30^,^18^,^29^

IPAQ = International Physical Activity Questionnaire; PA = Physical Activity; PACE+ = PACE + Adolescent Physical Activity Measure; PAQ-A = Physical Activity Questionnaire for Adolescents.

a The regional classification of countries is based on World Health Organization regions.

**Table 3:** Overview of the changes in PA and health-related behaviors.

	Increased	No changes	Decreased
Physical activity	^19^,^22^	–	^25^,^33^,^14^,^15^,^31^,^16^,^17^,^30^,^34^,^20^,^23^,^32^,^24^
Screen time	^25^,^31^,^16^,^17^,^34^,^19^,^20^,^22^,^32^		–
Sleep duration	^25^,^31^,^17^,^20^,^21^,^32^	^16^	–
Sedentary time	^31^,^32^	–	–

**Table 4:** Overview of the associations found with PA.

	Positive		None		Negative	
	During	Changes	During	Changes	During	Changes
Gender (ref: girls)	^26^,^15^,^30^,^19^,^23^	–	^29^	^25^,^17^,^28^	–	^33^,^23^
Age	–	–	^21^	^17^	^26^,^29^	^25^,^26^,^28^,^30^,^22^
BL-PAL, BL fitness ^a^	^33^,^18^,^23^,^24^	^33^	–	–	–	–
Home						
detached house	–	^25^,^28^	–	–	–	–
garden, garage	–	^17^	–	–	–	–
large spaces	–	^25^,^17^	–	–	–	–
dog ownership	^30^	–	–	–	–	–
number of families	–	^28^	–	^21^	–	–
family income	–	^28^	–	^25^,^26^	–	–
Urbanization, dwelling density	–	–	–	–	–	^25^,^28^,^24^
Parent						
education level	^15^	^17^	–	^28^	^21^	^25^
non-native, immigration	–	–	–	^26^	–	^17^
support, encouragement	^34^	–	–	–	–	–
stress, anxiety	–	–	–	–	^20^	^27^
Conflict	–	–	–	–	^15^	–

BL = baseline; PA = Physical Activity; PAL = PA level.

### Demographic characteristics

Many studies were conducted in Europe (n = 9) and the Americas (n = 8). The two Asian studies were both conducted in China. One study each was found in the Eastern Mediterranean and Western Pacific, but none were found for Africa. The age group of subjects ranged from preschool through adolescence. The number of samples ranged from less than 100 (in two studies) to more than 1000 (in nine studies), including two studies with more than 10,000 (both of which were from the same survey project). The data collection period was mostly from March to May 2020, although some studies were conducted in China from January to February 2020.

### PA measurement methods

Online surveys were used in all 21 studies. The Physical Activity Questionnaire for Adolescents (PAQ-A) and the International Physical Activity Questionnaire (IPAQ; long or short version) were frequently used in the surveys. Two studies measured PA with devices, one using tri-axial accelerometers and the other using smartphone sensors.

As for the survey of changes found in PA before and during the pandemic, eight studies compared baseline and follow-up data, and the other 13 studies were retrospective. Seven of the retrospective studies asked participants to recall their PA before the pandemic, while six studies asked participants to answer questions about the perceived changes in their PA between before and during the pandemic. One study collected qualitative data, asking adolescents to describe facilitators and inhibitors of PA during the pandemic.

### PA and health-related behaviors

The changes in children's PA and health-related behaviors are shown in Table 3. A significant decrease in PA was reported in 13 of the 21 studies, whereas two studies reported an increase in PA. Six other studies also reported a decrease in PA, although they were not statistically examined. In the studies that measured health-related behaviors, significant increases in screen time, sleep duration, and sedentary behaviors were generally reported.

### Factors related to PA

Factors associated with PA during the pandemic are shown in Table 4. Overall, older children or adolescents were more likely to reduce their PA during the pandemic than younger children. Boys had higher PA levels during the pandemic but were more likely to experience larger reductions in PA than girls. Children with higher baseline physical fitness were more likely to be active during the pandemic.

Children's living environments were also related to changes in PA during the pandemic. Children who lived in detached houses, houses with yards, garages, or more space and children from large families tended to experience lower decreases in PA, while decreases in PA were greater for children living in urban areas than children in rural areas.

Although studies reporting the effects of parental and family factors were limited, decreases in PA tended to be greater when parents were foreign nationals, had higher anxiety or stress levels, and had family conflicts. In contrast, parent's support and encouragement were positively related to children's PA. Findings related to associations between parents' education levels and PA are inconsistent, with some studies finding positive, negative, and no significant association with changes in PA.

## Discussion

### Characteristics of survey contexts and methods

Most of the countries extracted in this review are located in Europe and the Americas, where the number of infected people is considerable, and China, where the disease was first discovered and spread. Similar to the review of university students,^11^ most studies were conducted in high- or upper-middle-income countries. As developing countries have limited medical resources and are facing vaccination delays,^35^^,^^36^ a survey of the pandemic's impact on children is urgently needed even though the infection rate in such countries appears to be low.

Online surveys were used in all the studies included in the current review, likely because of the social restrictions issued during the pandemic that made on-site investigations difficult. Online surveys have allowed researchers to collect data from thousands of subjects in multiple countries. This trend was similar to that found in Caputo and Reichert's review.^10^

Although the IPAQ and PAQ-A have been verified as having acceptable reliability,^37^^,^^38^ there are concerns about the variability and reliability of the self-assessments conducted by the participants.^37^^,^^39^ In addition, 13 of the 21 studies were retrospective, asking respondents to recall their PA habits before the pandemic or discuss perceived changes in their PA. Concerns about recall bias exist for surveys that ask respondents to recall the time before the pandemic began. Moreover, studies in which respondents stated the perceived change in their PA was “less” or “much less,” etc., compared to before the pandemic could not evaluate the amount of PA they engaged in and may reflect a degree of subjectivity in the respondents. Since the spread of the infection was difficult to predict, possessing baseline data from before the pandemic is valuable. These available data are expected to be used for comparative studies.

Only one study used accelerometers to evaluate PA. However, it reported difficulties getting children to wear the devices long enough to ensure the reliability of the data.^19^ Smartphones, used in one study,^34^ do not require any special equipment, and tracking applications can collect data over time using a pedometer and GPS tracking. This method is expected to be used in future large-scale studies measuring adolescents’ PA during the pandemic. In fact, several studies have assessed PA in adults using smartphone app data during the pandemic.^40^^,^^41^ However, issues concerning measurement accuracy and user bias remain.^40^

### Changes in PA throughout the pandemic

Most studies reported a significant decrease in PA and changes in health-related behaviors during the pandemic, such as increased screen time, sedentary behaviors, and sleep duration. It should be noted that a decline in sleep quality was reported despite the increase in sleep duration.^25^ In addition to a decrease in PA, these lifestyle changes may critically impact one's physical and mental health. As López-Bueno warned in his narrative review, decreased PA, increased sedentary time, and screen exposure among children were confirmed.^42^ This review presents data on short-term changes in the first half of 2020, but lifestyle changes over an extended period would have more serious effects on children's health. The World Health Organization and Centers for Disease Control and Prevention are encouraging to engage in PA for maintaining physical and mental health together with taking infection prevention measures.^43^^,^^44^ Furthermore, there is evidence that regular PA can improve one's immune function and reduce the risk of viral infections.^45^^,^^46^ Given that lifestyle habits in childhood are to some extent still relevant in adulthood,^8^^,^^9^ proactive strategies are needed for “improving humankind's resiliency during future pandemics.”^47^^(p109^^)^

Overall, children's PA decreased during the pandemic, but two studies found that PA increased. A study of Swedish preschoolers showed an increase in exercise and outdoor playtime.^19^ A German study also showed an increase in overall PA due to an increase in habitual activities, despite a decrease in sports activity.^22^ Additionally, some studies described the features of children who increased or adjusted their PA. For instance, increases in PA at home, the neighborhood, and household activities were reported.^25^^,^^27^^,^^48^ One study also introduced the use of online exercise resources among adolescents.^26^ The study that conducted a qualitative analysis of the factors affecting PA reported that school closures were seen as a promoting factor as they created more time for PA.^18^ In addition to the current review, a study of college health science students also reported an increase in the students' PA levels during lockdown periods, which modified their habitual behaviors.^49^ A study of German adults also reported that subjects who maintained or increased their PA exercised at home and through outdoor endurance activities.^50^ These findings suggest that social restrictions do not necessarily obstruct PA but can maintain or increase PA by adjusting the location and form of exercise (i.e., in the neighborhood or at home) when the restrictions are mitigated.

### Age and gender differences

During the pandemic, the PA of boys and older children or adolescents was more likely to decrease. With increasing age, adolescents are known to become less physically active than children.^51^

The pandemic caused adolescents to be more inactive. This may be related to the fact that toddlers and preschool children are more likely to engage in play in small spaces at home or in their neighborhoods.^25^ This may also be because boys and adolescents are more likely to participate in organized team sports, most of which were canceled due to the pandemic.^23^ However, the decrease in PA among girls may not be statistically significant because girls generally have lower levels of PA.^23^ In fact, five out of six studies reported that girls were less physically active than boys during the pandemic. In other words, the results may not be optimistic for girls.

### Home and community environment

Previous studies on the effects of such residential environments on children's PA have produced limited research and inconsistent results^52^, ^53^, ^54^ since such residential environments may be related to diverse factors, such as climate, income, or racial and other sociometric factors. However, predictably, during periods in which stay-at-home orders were issued, PA was less likely to decrease in children who lived in detached houses, houses with a yard, garage, or larger spaces, and in rural areas; relatedly, the pandemic may have restricted PA for urban or apartment dwellers, especially in densely populated areas where space is limited and there was a large number of infected cases. The closure of sports facilities may have also had a greater impact on those in urban areas.^24^ However, children living in rural areas are generally less physically active than those living in urban areas.^55^^,^^56^ Therefore, it is possible that a statistically significant decrease did not appear in the PA of children in rural areas because their PA before the pandemic was lower than that of children living in urban areas.

### Parental factors

It has been consistently argued that parental support, encouragement, and engagement in PA are important facilitators of children's PA.^53^^,^^57^^,^^58^ However, immigrant children in the U.S. have been reported to have lower PA levels than native-born children.^59^^,^^60^ The current review indicates that parental stress and anxiety, non-native parents, and family conflicts are negatively related to children's PA, while parents' supportive encouragement is expected to help children maintain their PA during the pandemic. This means that the shortage of supportive environments for PA could create a larger gap among children during the pandemic. However, the results on parental education were not consistent. These findings validated López-Bueno's warning about the potential health risks for children from socio-economically disadvantaged families.^42^ Although studies have reported that parents with higher education levels tend to encourage their children to lead healthier lifestyles and engage in higher levels of PA,^53^^,^^61^ previous literature reviews have shown a negative association between higher maternal, rather than parental, education level and children's PA,^62^ typically in countries with lower economic status.^63^^,^^64^ The studies in this review do not provide a clear argument for the influence of parental educational background. However, families with highly educated mothers tend to have both parents working, and the absence of a caregiver during school closures may lead to a decline in PA.^25^

### Limitations

This review has several limitations. First, there may be a study bias. Since the review examines studies published in 2020, the survey period is limited to the first half of 2020. Longer-term effects may need to be investigated. The regions reviewed are also biased toward Europe and the Americas. Second, the status and degree of intensity and duration of COVID-19 restrictions differ between countries and regions. Finally, the quality of the studies was not evaluated. Because the survey contexts and methods used varied considerably, quality assessment and meta-analysis are left as issues for future investigation.

## Conclusions

This scoping review suggests that the COVID-19 pandemic has caused a decline in PA among children. The decrease was greater in boys, older children or adolescents, and those who live in apartments or houses with limited space, high population density, or urban areas. However, the studies suggest that PA could be maintained or increased by ensuring parental support and engagement, making effective use of available time, and arranging locations and forms of PA. Moreover, children and adolescents with higher levels of PA tended to cope better with barriers to PA. Since the effects of COVID-19 are predicted to be prolonged, public support for children and families should consider these findings to prevent the negative effects of physical inactivity among children. One pessimistic scenario is that worldwide PA levels will continue to decline and could be worse than before the pandemic.^47^ We need to take the disease as an opportunity to reaffirm the awareness of the importance of daily PA among children and adolescents as well as people of all generations.^46^^,^^47^ Moreover, given that globalized modern society cannot prevent the transmission of infectious diseases that might appear in the future, it is necessary to accumulate knowledge and prepare for rapid countermeasures to keep children active and healthy.

## Funding

This research did not receive any specific grant from funding agencies in the public, commercial, or not-for-profit sectors.

## Declaration of competing interest

The authors have no conflicts of interest relevant to this article.

## Figures and Tables

**Table A:** Summary of the characteristics and main findings of each study

Author, year, country	Survey period	Sample size, age^a^	Data collection		Main findings
			PA measurement	Comparison	
Aguilar-Farias (2020),^25^ Chile	Mar–Apr 2020	age 1–5, n = 3157	PAT (min/day), (online)	Recall	PAT significantly decreased (3.6–2.8 h/day); ST and SD increased. Older preschoolers had greater reductions in PA and SD but more increases in ST than toddlers (age 1). Children with space to play at home and living in rural areas had lower reductions in PA. Children whose caregivers had higher education levels and living in apartments had greater reductions in PA.
Delisle Nyström (2020),^19^ Sweden	Mar–May 2019 (BL), May–Jun 2020 (FU)	preschool (age 4.0 ± 0.5), n = 82	Tri-axial accelerometer (ActiGraph wGT3x-BT) (BL survey), PAT (min/day), (online)	BL-FU	PAT (+53 min/day), time spent outside on weekdays (+124 min/day) and weekends (+68 min/day), and ST (+30 min/day) significantly increased during the pandemic. Children meeting WHO PA, ST, and SD guidelines were 90.3%, 37.8%, and 62.5%, respectively.
Dunton (2020),^26^ USA	Apr–May 2020	age 5–13, n = 211	PAT (min/day), Perceived changes in frequency of PA (5-point), (online)	Perceived changes	Parents perceived children's PA had decreased and SB had increased. Older children (age 9–13) perceived greater reductions in PA and increases in SB. PA at home, in the garage, or on neighborhood streets increased during the pandemic. About a third of children used remote/streaming activity classes and lessons.
Elnaggar (2020),^33^ Saudi Arabia	N/A (before and during social distancing restrictions)	age 14–18, n = 63	BL fitness test, PAQ-A (online)	BL-FU	PAL among the total sample and boys reduced significantly during the pandemic (3.05–2.77, PAQ-A). PAL for boys was significantly higher than girls at BL; no gender difference at FU. PAL during the pandemic was determined by BL fitness for girls.
Francisco (2020),^14^ Italy, Spain, and Portugal	Mar–Apr 2020	age 3–18, n = 1480	PAT (min/day, 6-point), (online)	Recall	PAT significantly declined; psychological and behavioral symptoms, ST, and SD increased. Before quarantine, most children practiced 30–60 min/day of PA (33.1%); during quarantine, most children practiced <30min/day of PA (53.0%).
Gilic (2020),^15^ Croatia, Bosnia and Herzegovina	Jan 2020 (BL), Apr 2020 (FU)	age 15–18, n = 688	PAQ-A (online)	BL-FU	A significant decline in PAL was recorded between BL and FU (2.98–2.31, PAQ-A). Approximately 50% of adolescents had sufficient PA (<2.73) at BL; at FU, 24% had sufficient PA. Paternal education positively associated with PAL before and during the pandemic; conflicts with parents/family had negative effect.
Jia (2020),^31^ China	Apr–May 2020	age 19.8 ± 2.3, n = 10082	IPAQ-long form (online)	Recall	PAL decreased significantly during lockdown; SB, SD, and ST increased. BMI significantly increased (from 21.8 to 22.1) after lockdown.
López-Bueno (2020),^16^ Spain	Mar–May 2020	age 3–16, n = 860	PAT (min/week) (online)	Recall	Children's PAT decreased significantly (−102.4 min/week), and ST increased (+2.9 h/day) during confinement.
McCormack (2020),^27^ Canada	Apr–May 2020	age 5–17, n = 345	Perceived changes in frequency of PA (5-point) PA, Days of ≥60min MVPA/week, (online)	Perceived changes	Children achieved ≥60 min of MVPA on 3.48 ± 2.41 days/week (18.3% with no MVPA days). PA at home increased (48.8%) or remained unchanged (32.9%) during the pandemic, while PA outdoors either increased (38.7%) or decreased (39%). Higher anxiety parents reported more decreases in their child's PA and increases in ST.
Medrano (2020),^17^ Spain	Sep–Dec 2019 (BL), Mar–Apr 2020 (FU)	age 8–16, n = 291	PAL, PAT (min/day) (YAP), (online)	BL-FU	Total PA decreased significantly (−91 min/day), whereas ST and SD increased. PA decreases were higher in children with mothers of non-Spanish origin or non-university studies than their counterparts. Children with outside space at home or big spaces (garage, attic, or gym) had fewer decreases in PA.
Mitra (2020),^28^ Canada	Apr 2020	age 5–17, n = 1472	Perceived changes in frequency of PA (5-point), (online)	Perceived changes	Majority reported a decrease in outdoor activities (64%) and increases in indoor play (53%) and ST (79%). Youth (age 12–17) experienced a larger decline in PA than children (age 5–11). Living in a house (not an apartment), a low dwelling density, and further from major roads, and higher income and multi-child households were associated with increases in outdoor activities.
Moore (2020),^30^ Canada	Apr 2020	age 5–17, n = 1472	Perceived changes in frequency of PA (5-point), Days of ≥60 m MVPA/week (online)	Perceived changes	Significant declines in PA and outside time and increases in SB, ST, and SD during the pandemic were found. Younger parents were associated with less decline in child PA. Youths (age 12–17) experienced greater declines in PA than children (age 5–11). Parental encouragement and support, engagement in PA, and dog ownership were positively associated with PAL.
Munasinghe (2020),^34^ Australia	Nov 2019 (BL), Apr 2020 (FU)	age 13–19, n = 582	Smartphone sensors (Ethica app), PACE+ (days of ≥60 min PA/week), (online)	BL–FU (sequential)	Significant decrease in days with ≥60 min PA/day (OR = .53, CI = .34–.83), and steps/day, sensor-based activity time were estimated. Physical distancing was associated with lower levels of happiness, increases in ST and social media and internet use, and decreases in fast food consumption.
Ng (2020),^18^ Ireland	Apr 2020	age 12–18, n = 1214	PACE+ (days of ≥60 min PA/week), Perceived changes in frequency of PA (3-point), PA barriers and facilitators (open-ended), (online)	Perceived changes	Adolescents displayed less PA (50%), no change (30%), or more PA (20%) during lockdown. Adolescents with prior strong PA habits were over two times more likely to report more PA during lockdown than those with weak PA habits. The biggest barrier to PA was club training cancellations; adolescents who increased their PA stated that the school closure provided them more time for PA.
Orgilés (2020),^20^ Italy and Spain	N/A (before Jun 2020, submitted)	age 3–18, n = 1143	PAT (min/day), (online)	Recall	Children has significantly less PA, more ST, and longer SD during quarantine. Only 14.8% of youth undertook ≥60 min/day of PA, compared to before the quarantine (54.1%). Higher level of parental stress was related to more ST and less PAT and SD.
Ruíz-Roso (2020),^21^ Italy, Spain, Brazil, Chile, and Colombia	Apr–May 2020	age 10–19, n = 726	IPAQ-long form, (online)	Recall	The proportion of adolescents considered inactive was 73.0% before social isolation and 79.5% during. Adolescents in Latin America and those whose mothers have high levels of education were less active during lockdown.
Sá (2020),^29^ Brazil	Mar 2020	age 0–12, n = 816	Perceived changes in frequency of PA (5-point), PAT (h/day), (online)	Perceived changes	Most parents reported reductions in PA among their children (much less: 46.1%; less: 37.0%) and increases in ST and family activities during isolation.
Schmidt (2020),^22^ Germany	Aug 2018–Mar 2020 (BL), Apr–May 2020 (FU)	age 4–17, n = 1711	MoMo-PAQ (28 items for PAL), (online)	BL-FU	Days with ≥60 min of PA significantly increased (+0.44 day/week). Total PA increased, associated with increased habitual activities, but participation in sport activities declined. Adolescents (age 14–17) saw a lower increase in PA and a greater increase in ST than younger children.
Sekulic (2020),^23^ Croatia	Sep–Oct 2019 (fitness test), Feb 2020 (PAL-BL), Apr 2020 (PAL-FU)	age 15–18, n = 388	BL fitness test, PAQ-A, (online)	BL-FU	PAL significantly decreased in total sample (2.99–2.67, PAQ-A) and boys (3.10–2.79); no differences were found in girls (2.71–2.59). Youth with higher BL fitness were positively correlated with higher PAL during social distancing.
Yang (2020),^32^ China	Jan–Feb 2020	age 19.8 ± 2.3, n = 10082	IPAQ-long form, (online)	Recall	Frequency of PA significantly decreased, while SB, SD, and ST increased. BMI significantly increased during lockdown in the overall youth sample (21.8–22.6). Overweight/obese children have significantly increased (21.3%–25.1%).
Zenic (2020),^24^ Croatia	Sep–Oct. 2019 (fitness test) Mar 2020 (PAL-BL), Apr 2020 (PAL-FU)	age 16.5 ± 2.1, n = 823	BL fitness test, PAQ-A, (online)	BL-FU	PAL decreased significantly in the total sample (2.97–2.63, PAQ-A). Adolescents in urban areas had a greater decrease in PAL (3.11–2.68, PAQ-A) than those in rural areas. The BL fitness of urban adolescents predicted their PAL during the pandemic; no evidence for the same was found among rural adolescents.

a The age of samples is given as the age of the children; in some studies, however, parents were asked to evaluate the PA levels for younger children.

BL = baseline; BMI = body mass index; CI = confidence interval; FU = follow-up; IPAQ = International Physical Activity Questionnaire; MoMo-PAQ = MoMo physical activity questionnaire; MVPA = moderate-to-vigorous physical activity.

OR = odds ratio; PACE+ = PACE + Adolescent Physical Activity Measure; PAL = PA level; PAQ-A = Physical Activity Questionnaire for Adolescents; PAT = PA time; SB = sedentary behavior; SD = sleep duration; ST = screen time; YAP = Youth Activity Profile questionnaire.

## References

[1] UNESCO. UNESCO (2020). Figures show Two thirds of an academic year lost on average worldwide due to Covid-19 closures UNESCO. https://en.unesco.org/news/unesco-figures-show-two-thirds-academic-year-lost-average-worldwide-due-covid-19-school.

[2] Xie X., Xue Q., Zhou Y. (2020). Mental health status among children in home confinement during the coronavirus disease 2019 outbreak in Hubei Province, China. JAMA Pediatr.

[3] Duan L., Shao X., Wang Y. (2020). An investigation of mental health status of children and adolescents in China during the outbreak of COVID-19. J Affect Disord.

[4] Bruni O., Malorgio E., Doria M. (2021). Changes in sleep patterns and disturbances in children and adolescents in Italy during the Covid-19 outbreak. Sleep Med.

[5] Chen F., Zheng D., Liu J., Gong Y., Guan Z., Lou D. (2020). Depression and anxiety among adolescents during COVID-19: a cross-sectional study. Brain Behav Immun.

[6] Rundle A.G., Park Y., Herbstman J.B., Kinsey E.W., Wang Y.C. (2020). COVID-19–Related school closings and risk of weight gain among children. Obesity.

[7] World Health Organization (2020). Physical activity. https://www.who.int/news-room/fact-sheets/detail/physical-activity.

[8] Malina R.M. (2001). Physical activity and fitness: pathways from childhood to adulthood. Am J Hum Biol.

[9] Reilly J.J., Kelly J. (2011). Long-term impact of overweight and obesity in childhood and adolescence on morbidity and premature mortality in adulthood: systematic review. Int J Obes.

[10] Caputo E.L., Reichert F.F. (2020). Studies of physical activity and COVID-19 during the pandemic: a scoping review. J Phys Activ Health.

[11] López-Valenciano A., Suárez-Iglesias D., Sanchez-Lastra M.A., Ayán C. (2021). Impact of COVID-19 pandemic on university students' physical activity levels: an early systematic review. Front Psychol.

[12] Arksey H., O'Malley L. (2005). Scoping studies: towards a methodological framework. Int J Soc Res Methodol Theory Pract.

[13] Tricco A.C., Lillie E., Zarin W. (2018). PRISMA extension for scoping reviews (PRISMA-ScR): checklist and explanation. Ann Intern Med.

[14] Francisco R., Pedro M., Delvecchio E. (2020). Psychological symptoms and behavioral changes in children and adolescents during the early phase of COVID-19 quarantine in three European countries. Front Psychiatr.

[15] Gilic B., Ostojic L., Corluka M., Volaric T., Sekulic D. (2020). Contextualizing parental/familial influence on physical activity in adolescents before and during COVID-19 pandemic: a prospective analysis. Children.

[16] López-Bueno R., López-Sánchez G.F., Casajús J.A. (2020). Health-related behaviors among school-aged children and adolescents during the Spanish covid-19 confinement. Front Pediatr.

[17] Medrano M., Cadenas-Sanchez C., Oses M., Arenaza L., Amasene M., Labayen I. (2020). Changes in lifestyle behaviours during the COVID-19 confinement in Spanish children: a longitudinal analysis from the MUGI project. *Pediatr Obes*. Published online.

[18] Ng K., Cooper J., McHale F., Clifford J., Woods C. (2020). Barriers and facilitators to changes in adolescent physical activity during COVID-19. BMJ Open Sport Exerc Med.

[19] Delisle Nyström C., Alexandrou C., Henström M. (2020). International study of movement behaviors in the early years (SUNRISE): results from SUNRISE Sweden's pilot and COVID-19 study. Int J Environ Res Publ Health.

[20] Orgilés M., Morales A., Delvecchio E., Mazzeschi C., Espada J.P. (2020). Immediate psychological effects of the COVID-19 quarantine in youth from Italy and Spain. Front Psychol.

[21] Ruíz-Roso M.B., de Carvalho Padilha P., Matilla-Escalante D.C. (2020). Changes of physical activity and ultra-processed food consumption in adolescents from different countries during covid-19 pandemic: an observational study. Nutrients.

[22] Schmidt S.C.E., Anedda B., Burchartz A. (2020). Physical activity and screen time of children and adolescents before and during the COVID-19 lockdown in Germany: a natural experiment. Sci Rep.

[23] Sekulic D., Blazevic M., Gilic B., Kvesic I., Zenic N. (2020). Prospective analysis of levels and correlates of physical activity during COVID-19 pandemic and imposed rules of social distancing; gender specific study among adolescents from Southern Croatia. Sustain Times.

[24] Zenic N., Taiar R., Gilic B. (2020). Levels and changes of physical activity in adolescents during the COVID-19 pandemic: contextualizing urban vs. Rural living environment. Appl Sci.

[25] Aguilar-Farias N., Toledo-Vargas M., Miranda-Marquez S. (2020). Sociodemographic predictors of changes in physical activity, screen time, and sleep among toddlers and preschoolers in Chile during the COVID-19 pandemic. Int J Environ Res Publ Health.

[26] Dunton G.F., Do B., Wang S.D. (2020). Early effects of the COVID-19 pandemic on physical activity and sedentary behavior in children living in the U.S. BMC Publ Health.

[27] McCormack G.R., Doyle-Baker P.K., Petersen J.A., Ghoneim D. (2020). Parent anxiety and perceptions of their child's physical activity and sedentary behaviour during the COVID-19 pandemic in Canada. Prev Med Reports.

[28] Mitra R., Moore S.A., Gillespie M. (2020). Healthy movement behaviours in children and youth during the COVID-19 pandemic: exploring the role of the neighbourhood environment. Health Place.

[29] Cdsc de Sá, Pombo A., Luz C., Rodrigues L.P., Cordovil R. (2020). Covid-19 social isolation in Brazil: effects on the physical activity routine of families with children. Rev Paul Pediatr.

[30] Moore S.A., Faulkner G., Rhodes R.E. (2020). Impact of the COVID-19 virus outbreak on movement and play behaviours of Canadian children and youth: a national survey. Int J Behav Nutr Phys Activ.

[31] Jia P., Zhang L., Yu W. (2020). Impact of COVID-19 lockdown on activity patterns and weight status among youths in China: the COVID-19 Impact on Lifestyle Change Survey (COINLICS). *Int J Obes*. Published online December.

[32] Yang S., Guo B., Ao L. (2020). Obesity and activity patterns before and during COVID -19 lockdown among youths in China. Clin Obes.

[33] Elnaggar R.K., Alqahtani B.A., Mahmoud W.S., Elfakharany M.S. (2020). Physical activity in adolescents during the social distancing policies of the COVID-19 pandemic. Asia Pac J Publ Health.

[34] Munasinghe S., Sperandei S., Freebairn L. (2020). The impact of physical distancing policies during the COVID-19 pandemic on health and well-being among Australian adolescents. J Adolesc Health.

[35] Campaigners warn that 9 out of 10 people in poor countries are set to miss out on COVID-19 vaccine next year | Amnesty International. https://www.amnesty.org/en/latest/news/2020/12/campaigners-warn-that-9-out-of-10-people-in-poor-countries-are-set-to-miss-out-on-covid-19-vaccine-next-year/.

[36] World's most vulnerable countries lack the capacity to respond to a global pandemic credit: MFD/elyas alwazir | office of the high representative for the least developed countries, landlocked developing countries and small island developing states. https://www.un.org/ohrlls/news/worlds-most-vulnerable-countries-lack-capacity-respond-global-pandemic-credit-mfdelyas-alwazir.

[37] Helmerhorst H.J.F., Brage S., Warren J., Besson H., Ekelund U. (2012). A systematic review of reliability and objective criterion-related validity of physical activity questionnaires. Int J Behav Nutr Phys Activ.

[38] Craig C.L., Marshall A.L., Sjöström M. (2003). International physical activity questionnaire: 12-Country reliability and validity. Med Sci Sports Exerc.

[39] Timperio A., Salmon J., Crawford D. (2003). Validity and reliability of a physical activity recall instrument among overweight and non-overweight men and women. J Sci Med Sport.

[40] McCarthy H., Potts H., Fisher A. (2020). Title: physical activity behaviour before, during and after COVID-19 restrictions: a longitudinal smartphone tracking study of 5395 UK adults. (Preprint). J Med Internet Res.

[41] Tison G.H., Avram R., Kuhar P. (2020). Worldwide effect of COVID-19 on physical activity: a descriptive study. Ann Intern Med.

[42] López-Bueno R., López-Sánchez G.F., Casajús J.A., Calatayud J., Tully M.A., Smith L. (2020). Potential health-related behaviors for pre-school and school-aged children during COVID-19 lockdown: a narrative review. Prev Med.

[43] World Health Organization (2020). Stay physically active during self-quarantine. Worl Health Organization (WHO). https://www.euro.who.int/en/health-topics/health-emergencies/coronavirus-covid-19/publications-and-technical-guidance/noncommunicable-diseases/stay-physically-active-during-self-quarantine.

[44] Centers for Disease Control and Prevention (2020). Physical activity basics. Physical activity. https://www.cdc.gov/physicalactivity/basics/index.htm.

[45] Laddu D.R., Lavie C.J., Phillips S.A., Arena R. (2021). Physical activity for immunity protection: inoculating populations with healthy living medicine in preparation for the next pandemic. Prog Cardiovasc Dis.

[46] Arena R., Lavie C.J. (2021). The global path forward – healthy living for pandemic event protection (HL – PIVOT). Prog Cardiovasc Dis.

[47] Hall G., Laddu D.R., Phillips S.A., Lavie C.J., Arena R. (2021). A tale of two pandemics: how will COVID-19 and global trends in physical inactivity and sedentary behavior affect one another?. Prog Cardiovasc Dis.

[48] Mitra R., Moore S.A., Gillespie M. (2020). Healthy movement behaviours in children and youth during the COVID-19 pandemic: exploring the role of the neighbourhood environment. Health Place.

[49] Romero-Blanco C., Rodríguez-Almagro J., Onieva-Zafra M.D., Parra-Fernández M.L., Prado-Laguna M.D.C., Hernández-Martínez A. (2020). Physical activity and sedentary lifestyle in university students: changes during confinement due to the covid-19 pandemic. Int J Environ Res Publ Health.

[50] Mutz M., Gerke M. (2020). Sport and exercise in times of self-quarantine: how Germans changed their behaviour at the beginning of the Covid-19 pandemic. Int Rev Sociol Sport.

[51] Dumith S.C., Gigante D.P., Domingues M.R., Kohl H.W. (2011). Physical activity change during adolescence: a systematic review and a pooled analysis. Int J Epidemiol.

[52] Davison K.K., Lawson C.T. (2006). Do attributes in the physical environment influence children's physical activity? A review of the literature. Int J Behav Nutr Phys Activ.

[53] Ferreira I., van der Horst K., Wendel-Vos W., Kremers S., van Lenthe F.J., Brug J. (2007). Environmental correlates of physical activity in youth ? a review and update. Obes Rev.

[54] Maitland C., Stratton G., Foster S., Braham R., Rosenberg M. (2013). A place for play? The influence of the home physical environment on children's physical activity and sedentary behaviour. Int J Behav Nutr Phys Activ.

[55] Regis M.F., Oliveira L.M., Santos A.R., Leonidio A.C., Diniz P.R., Freitas C.M. (2016). Urban versus rural lifestyle in adolescents: associations between environment, physical activity levels and sedentary behavior. Einstein (Sao Paulo).

[56] Drenowatz C., Hinterkörner F., Greier K. (2020). Physical fitness in upper Austrian children living in urban and rural areas: a cross-sectional analysis with more than 18,000 children. Int J Environ Res Publ Health.

[57] Edwardson C.L., Gorely T. (2010). Parental influences on different types and intensities of physical activity in youth: a systematic review. Psychol Sport Exerc.

[58] Østbye T., Malhotra R., Stroo M. (2013). The effect of the home environment on physical activity and dietary intake in preschool children. Int J Obes.

[59] Kimbro R.T., Kaul B. (2016). Physical activity disparities between US-born and immigrant children by maternal region of origin. J Immigr Minority Health.

[60] Singh G.K., Yu S.M., Siahpush M., Kogan M.D. (2008). High levels of physical inactivity and sedentary behaviors among US immigrant children and adolescents. Arch Pediatr Adolesc Med.

[61] Muñoz-Galiano I.M., Connor J.D., Gómez-Ruano M.A., Torres-Luque G. (2020). Influence of the parental educational level on physical activity in schoolchildren. Sustainability.

[62] Stalsberg R., Pedersen A.V. (2010). Effects of socioeconomic status on the physical activity in adolescents: a systematic review of the evidence. Scand J Med Sci Sports.

[63] Muthuri S.K., Onywera V.O., Tremblay M.S. (2016). Relationships between parental education and overweight with childhood overweight and physical activity in 9-11 year old children: results from a 12-country study. PloS One.

[64] Muthuri S.K., Wachira L.J.M., Leblanc A.G. (2014). Temporal trends and correlates of physical activity, sedentary behaviour, and physical fitness among school-aged children in Sub-Saharan Africa: a systematic review. Int J Environ Res Publ Health.

